# Global genetic deletion of Ca_V_3.3 channels facilitates anaesthetic induction and enhances isoflurane-sparing effects of T-type calcium channel blockers

**DOI:** 10.1038/s41598-020-78488-8

**Published:** 2020-12-09

**Authors:** Simon Feseha, Tamara Timic Stamenic, Damon Wallace, Caesare Tamag, Lingling Yang, Jen Q. Pan, Slobodan M. Todorovic

**Affiliations:** 1 Department of Anesthesiology, University of Colorado, Anschutz Medical Campus, Mail Stop 8130, 12801 E. 17th Avenue, Rm L18-4100, Aurora, CO 80045 USA; 2 Neuroscience, University of Colorado, Anschutz Medical Campus, Aurora, 80045 USA; 3 Pharmacology Graduate Programs, University of Colorado, Anschutz Medical Campus, Aurora, 80045 USA; 4 Stanley Center for Psychiatric Research, Broad Institute of Harvard and MIT, Cambridge, USA

**Keywords:** Neuroscience, Medical research

## Abstract

We previously documented that the Ca_V_3.3 isoform of T-type calcium channels (T-channels) is inhibited by clinically relevant concentrations of volatile anaesthetics, including isoflurane. However, little is understood about the functional role of Ca_V_3.3 channels in anaesthetic-induced hypnosis and underlying neuronal oscillations. To address this issue, we used Ca_V_3.3 knock-out (KO) mice and a panselective T-channel blocker 3,5-dichloro-*N*-[1-(2,2-dimethyltetrahydro-pyran-4-ylmethyl)-4-fluoro-piperidin-4-ylmethyl]-benzamide (TTA-P2). We found that mutant mice injected with the vehicle showed faster induction of hypnosis than wild-type (WT) mice, while the percent isoflurane at which hypnosis and immobility occurred was not different between two genotypes. Furthermore, we found that TTA-P2 facilitated isoflurane induction of hypnosis in the Ca_V_3.3 KO mice more robustly than in the WT mice. Isoflurane-induced hypnosis following injections of TTA-P2 was accompanied with more prominent delta and theta EEG oscillations in the mutant mice, and reached burst-suppression pattern earlier when compared to the WT mice. Our findings point to a relatively specific value of Ca_V_3.3 channels in anaesthetic induced hypnosis. Furthermore, we propose that T-channel blockers may be further explored as a valuable adjunct to reducing the usage of potent volatile anaesthetics, thereby improving their safety.

## Introduction

It is known that general anaesthetics (GAs) induce sedation/hypnosis by targeting neuronal γ-aminobutyric acid type A (GABA_A_) and *N*-methyl-d-aspartate (NMDA) receptors^1^, as well as some voltage-gated ion channels^1–3^. In particular, the unique properties of T-type voltage-gated calcium channels (T-channels) are seemingly fitting for the regulation of neuronal excitability because they activate at low voltages, causing an influx of calcium ions. Molecular studies have shown that the pore forming α1 subunit of T-channels consist of three isoforms such as Ca_V_3.1, Ca_V_3.2, and Ca_V_3.3 with distinct pharmacological and kinetic properties^4^. These isoforms are differentially expressed in the thalamocortical circuits, which play an essential role in natural sleep and anaesthetic-induced hypnosis. For example, the Ca_V_3.1 isoform is widely expressed in the thalamocortical (TC) projection neurons, while Ca_V_3.2 and Ca_V_3.3 isoforms are concentrated in the nucleus reticularis thalami (nRT)^5^. The nRT is anatomically positioned as an optimal site to monitor cortical sensory processing and the regulation of the thalamus^6^ and is primarily comprised of inhibitory GABAergic neurons^7,8^. Additionally, optogenetic activation of the nRT facilitates a reduced arousal state, further validating the regulatory nature of the nRT^9^. Importantly, nRT possess an intrinsic firing ability mediated by Ca_V_3.3 channels expressed predominantly in the dendritic branches that mediate characteristic slowly inactivating T-currents^8,10–12^. We have previously established that both native thalamic and recombinant Ca_V_3.3 currents are inhibited by clinically relevant concentrations of volatile GAs including isoflurane^12,13^, but studies to date have not specifically evaluated the role of Ca_V_3.3 channels in anaesthetic mechanisms in vivo. Hence, we used mouse genetics and a selective pharmacological antagonist to investigate the role of Ca_V_3.3 channels in isoflurane-induced hypnosis and underlying thalamocortical oscillations.

## Materials and methods

### Animals

Experimental procedures with animals were performed according to the guidelines approved by the Institutional Animal Care and Use Committee (IACUC) of the University of Colorado Anschutz Medical Campus. Treatments of animals adhered to guidelines set forth in the NIH Guide for the Care and Use of Laboratory Animals*.* Our study was approved by the ethics committee of the University of Colorado Anschutz Medical Campus. Equal numbers (n = 10 mice in each cohort) of age-matched wild-type adult C57BL/6J (WT) and Ca_V_3.3 KO mice of both sexes (between 2 and 4 months of age) were used for behavioral experiments whenever possible. C57BL/6J mice were obtained from the Jackson laboratory (USA) while mice with global deletion of Ca_V_3.3 channel (α1I null) and Ca_V_3.1 channel (α1G null) were generated using C57BL/6J background as recently described elsewhere^3,14^. All animals were maintained on a 14/10 h light–dark cycle with food and water ad libitum. We also monitored physiological parameters with thermometer and pulse oximeter to assure normothermia and normal oxygenation of animals, respectively.

### Loss of righting reflex (LORR) and loss or withdrawal reflex (LOWR)

LORR is assessed by placing the mouse on its back until animal loses righting reflex. The criterion for the LORR is failure of mouse to right within a 30-s period. For LOWR, an alligator clip covered with airway tubing was used on proximal 1/3 tail and LOWR was considered when there was no withdrawal for a minimum of 30-s. After a 15-min wait period post i.p. injection, mice were placed on heating pad in the chamber with isoflurane equilibrated at 0.5% and 0.8% for LORR and LOWR experiments, respectively. Isoflurane then was increased by 0.1% every 10 min until LORR or LOWR was obtained.

### Anaesthetic induction

Induction time was assessed by measuring the time to LORR (TTLORR) at a constant concentration of 1.2% isoflurane. Mice were injected with the vehicle or TTA-P2 and placed on the heating pad in aneshetic chamber that was set at 1.2% isoflurane after a 30-min wait. Successful induction was determined when a mouse failed to right within a 30-s period.

### EEG data acquisition, 70% burst suppression, and spectral analysis

We used similar methods as we reported elsewhere^3^. Synchronized, time-locked video and electroencephalogram (EEG) signals were recorded using the Pinnacle system (Pinnacle Technology Inc., Lawrence, KS, USA). The EEG signals were amplified (100x) and digitized at a sampling frequency rate of 2000 Hz (high pass filter 0.5 Hz and low pass filter 500 Hz) and stored on a hard disk for offline analysis. The electrodes (one depth coated tungsten [anteroposterior—AP: − 1.35 mm, mediolateral—MD: 0 and dorsoventral—DV: − 3.6 mm], and two screw-type cortical [AP: − 1 mm, MD: ± 3 mm, DV: 0]) were implemented under continuous 1–1.5% isoflurane anaesthesia. Banamine–Merck (i.p. 2.5 mg/kg) was applied immediately after surgery and then every 24 h for 48 h. Seven to ten days after surgery, mice of both strains (7 WT and 9 Ca_V_3.3 KO) were placed in recording chamber and baseline EEG signals were recorded for 15 min before i.p. application of vehicle (15% 2-hydroxypropyl-β-cyclodextrin) or 60 mg/kg TTA-P2. Fifteen minutes after TTA-P2 or vehicle injection mice were placed in 0.6% or 1.0% of isoflurane-equilibrated chamber, respectively. Isoflurane was increased by 0.2% every 5 min until a 70% Burst Suppression Ratio (BSR) was evident.

Suppression was defined by at least a 70% decrease in amplitude (nearly isoelectric period) that lasted for a continuous 0.5 s period. The percentage of isoflurane at 70% BSR was determined by identifying the first 30 s period where 70% of the time (21 s) the aforementioned criteria for suppression was met. Analysis for BSR was performed by an investigator who was blinded for experimental conditions.

To compare spectra, 5 min of signal under vehicle and during TTA-P2 were extracted from 25–30 min of recordings. Local field potentials (LFPs) from the central medial region of the thalamus were recorded simultaneously, but were not analysed for this study. All spectral analysis was carried out using LabChart software version 8.1.16 (https://www.adinstruments.com/products/labchart/versions-and-licenses) and Origin software version 2018b (https://www.originlab.com/2018). The relative (%) power is calculated for different frequency ranges: δ (0.5–4 Hz), θ (4–8 Hz), α (8–13 Hz), β (13–30 Hz) and low γ (30–50 Hz). Additionally, power density (μV^2^/Hz) and spectrogram for the entire frequency range (0.5–50 Hz) were analysed.

Once experiments were completed, mice were anesthetized with ketamine (100 mg/kg) and electrolytic lesions were made by passing 5 μA current for 1 s (5 times). Mice were also anesthetized with isoflurane and perfused with ice-cold 0.1 M phosphate buffer containing 1% of potassium-ferrocyanide. Brains were extracted, kept in 4% formalin (PFA) for 2 days and sliced (100–150 μm) using a vibrating micro slicer (Leica VT 1200S). Images of coronal slices with electrode location conformation were obtained using bright-field Zeiss stereoscope and Zen Blue software.

### Drugs

We used similar methods as we reported elsewhere^3^. Isoflurane was purchased from McKesson (San Francisco, CA), 2-hydroxypropyl-β-cyclodextrin solution from Santa Cruz Biotechnology (Dallas, TX), ketamine and Banamine (Merck) were obtained from the University of Colorado Hospital pharmacy. TTA-P2 was purchased from Alomone Labs (Jerusalem, Israel). 3β-OH was obtained from Dr. Doug Covey (see^15^). All other compounds were purchased from Sigma Chemical (St. Louis, MO). For the EEG recordings and behavioral experiments, TTA-P2 was dissolved/suspended in 15% of 2-hydroxypropyl-β-cyclodextrin solution (diluted in saline) and injected intra-peritoneally (i.p.). 3β-OH was dissolved/suspended in 25% of 2-hydroxypropyl-β-cyclodextrin solution (diluted in Baxter water) and injected i.p.

### Data analysis

We used similar methods as we reported elsewhere^3^. In every experiment, we attempted to minimize the number of animals used. All animals with complete data set and physiological parameters were included in the study. Statistical analysis was performed using one-way and two-way repeated measure (RM) ANOVA as well as student unpaired and paired two-tailed *t*-test, where appropriate. We used *Bonferroni’s multiple comparisons test* where interaction between factors after one-way or two-way RM ANOVA was significant. Significance was accepted with *p* values < 0.05. Statistical and graphical analysis was performed using GraphPad Prism 8.00 software (GraphPad Software, La Jolla, CA, USA) and Origin 2018 (OriginLab, Northampton, MA, USA). EEG signals were analyzed using LabChart 8 (AD Instruments, Dunedin, New Zealand).

## Results

### Different effects of global deletion of Ca_V_3.3 isoform on isoflurane induced hypnosis and immobilization

**Figure 1:**
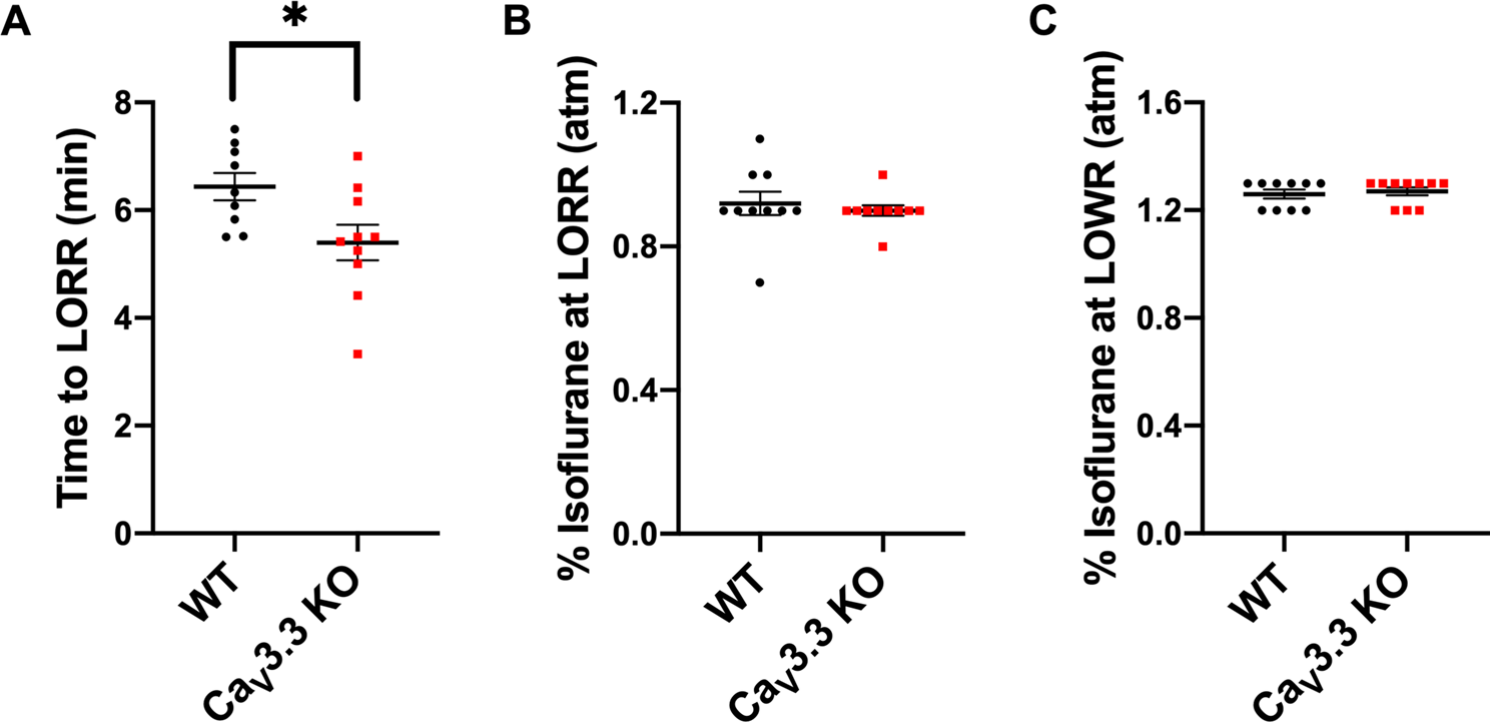
Ca_V_3.3. KO mice display faster induction time, but have similar requirement for hypnotic and immobilizing effects of isoflurane when compared to the WT mice. (**A**) Time of induction at 1.2% isoflurane for the WT versus Ca_V_3.3 KO mice with 15% cyclodextrin (vehicle). Mutant mice had a faster induction time in comparison to the WT mice (unpaired two-tailed t-test: t_17_ = 2.468, *p = 0.025). (**B**) Percent isoflurane at LORR for the WT versus Ca_V_3.3 KO mice with 15% cyclodextrin. No significant difference was identified between two cohorts (unpaired two-tailed t-test: t_18_ = 0.557, *p* = 0.584). (**C**) Percent isoflurane at LOWR for the WT versus Ca_V_3.3 KO mice with 15% cyclodextrin. There was no significant difference in % isoflurane required to reach LOWR between the WT and Ca_V_3.3 KO mice (unpaired two-tailed t-test: t_18_ = 0.447, *p* = 0.660).

We first set out to compare different anaesthesia endpoints between the WT and Ca_V_3.3 KO mice. We found that mutant mice had moderately faster TTLORR by about 15% when compared to the WT mice (Fig. 1A). In contrast, LORR per se and LOWR were not significantly affected in mutant mice when compared to the WT mice (Fig. 1B, C respectively). Hence, we conclude that isoflurane induction is faster in mutant mice while hypnosis and immobilization remained unchanged.

### TTA-P2 facilitates isoflurane induction in WT and Ca_V_3.3 KO mice

**Figure 2:**
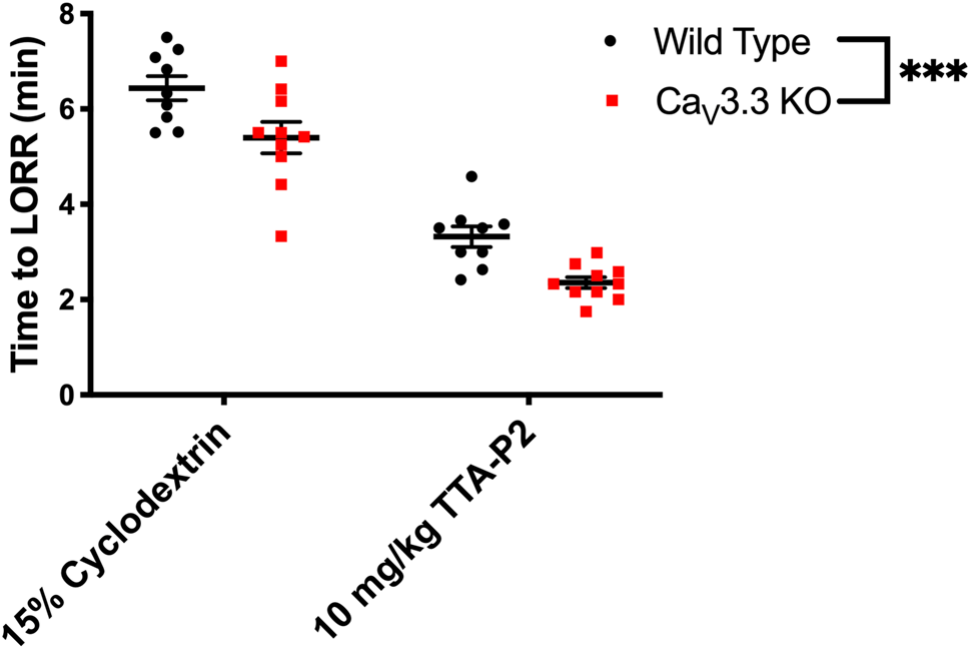
Selective pharmacological inhibition of T-channels with TTA-P2 facilitates anesthetic induction with isoflurane in the WT and mutant mice. Both WT and Ca_V_3.3 KO mice were injected with vehicle first (data from Fig. 1A) or TTA-P2 on different day and placed in a chamber set at 1.2% isoflurane after a 30-min wait period. Successful induction was determined when a mouse failed to right within a 30-s period. Note that both cohorts demonstrated a significant treatment difference (two-way RM ANOVA: F_1,17_ = 127.40, *p* < 0.001). However, the Ca_V_3.3 KO mice had overall faster induction times when compared to the WT group (two-way repeated measure (RM) ANOVA: F_1,17_ = 23.87, ***p < 0.001).

To our knowledge, there have been no previous studies investigating the use of TTA-P2, a panselective T-channel blocker^16^ on anaesthetic end points in vivo. We found that both WT and mutant mice were more easily induced by isoflurane pretreated with 10 mg/kg of TTA-P2. Specifically, there was up to 50% reduction of TTLORR with TTA-P2 being more potent in the mutant mice when compared to the WT mice (Fig. 2). Our results reveal for the first time the potential utility of TTA-P2 as an adjuvant to isoflurane in the context of induction, as this is a critical aspect of clinical anaesthesia.

### Injections of TTA-P2 decrease the requirement of isoflurane-induced hypnosis in both WT and Ca_V_3.3 KO mice in a dose-dependent manner

**Figure 3:**
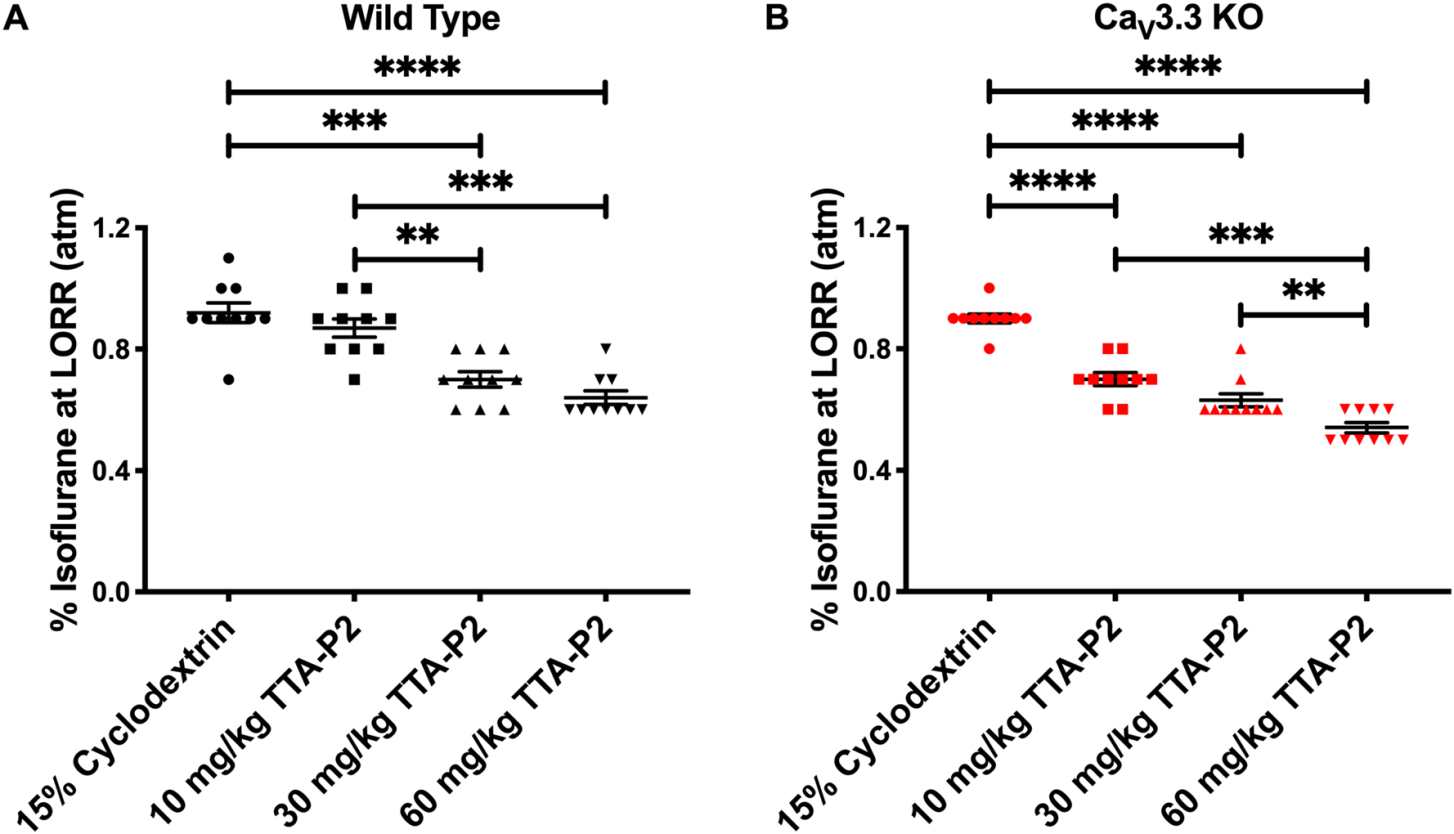
Dose-dependent sparing effect of TTA-P2 on isoflurane-induced hypnosis in the mutant and WT mice. (**A**) Percent isoflurane at LORR for WT mice. Data from WT mice injected with vehicle in Fig. 1B is used here as baseline. WT mice have a dose dependent decrease in the % isoflurane as the dose of TTA-P2 was escalated (one-way ANOVA: F_3,36_ = 29.14, *p* < 0.001). Bonferroni multiple comparison’s test further elucidated significant differences between 15% cyclodextrin and 30 mg/kg TTA-P2 (*p* < 0.001), 15% cyclodextrin and 60 mg/kg TTA-P2 (*p* < 0.001), 10 mg/kg TTA-P2 and 30 mg/kg TTA-P2 (*p* = 0.007), and 10 mg/kg TTA-P2 and 60 mg/kg TTA-P2 (*p* = 0.001). (**B**) Percent isoflurane at LORR for Ca_V_3.3 KO cohort. Data from mutant mice injected with vehicle in Fig. 1B is used here as control. Mutant mice had a dose dependent decrease in percent isoflurane for LORR as the dose of TTA-P2 increased (one-way ANOVA: F_3,36_ = 115.50, *p* < 0.001). Bonferroni multiple comparison’s test further identified significant differences between 15% cyclodextrin and 10 mg/kg TTA-P2 (*p* < 0.001), 15% cyclodextrin and 30 mg/kg TTA-P2 (*p* < 0.001), 15% cyclodextrin and 60 mg/kg TTA-P2 (*p* < 0.001), 10 mg/kg TTA-P2 and 60 mg/kg TTA-P2 (*p* < 0.001), 30 mg/kg TTA-P2 and 60 mg/kg TTA-P2 (*p* = 0.004).

We next examined the effects of i.p. injections of TTA-P2 at three incremental doses (10, 30, and 60 mg/kg) when combined with isoflurane. Because we found no significant difference between sexes in any of the three measures of anaesthetic potency, males and females were then grouped together for the remainder of the LORR analysis (Supplemental Figs. 1–2). In the evaluation of LORR, we found that both WT and Ca_V_3.3 KO demonstrated a marked dose-dependent decrease in requirement of isoflurane-induced hypnosis for up to about 30% (Fig. 3A, B). Together, these data reveal a dose-dependent facilitation of isoflurane-induced hypnosis with TTA-P2.

### TTA-P2 induced more prominent slow thalamocortical oscillations in the Ca_V_3.3 KO mice than in the WT mice

**Figure 4:**
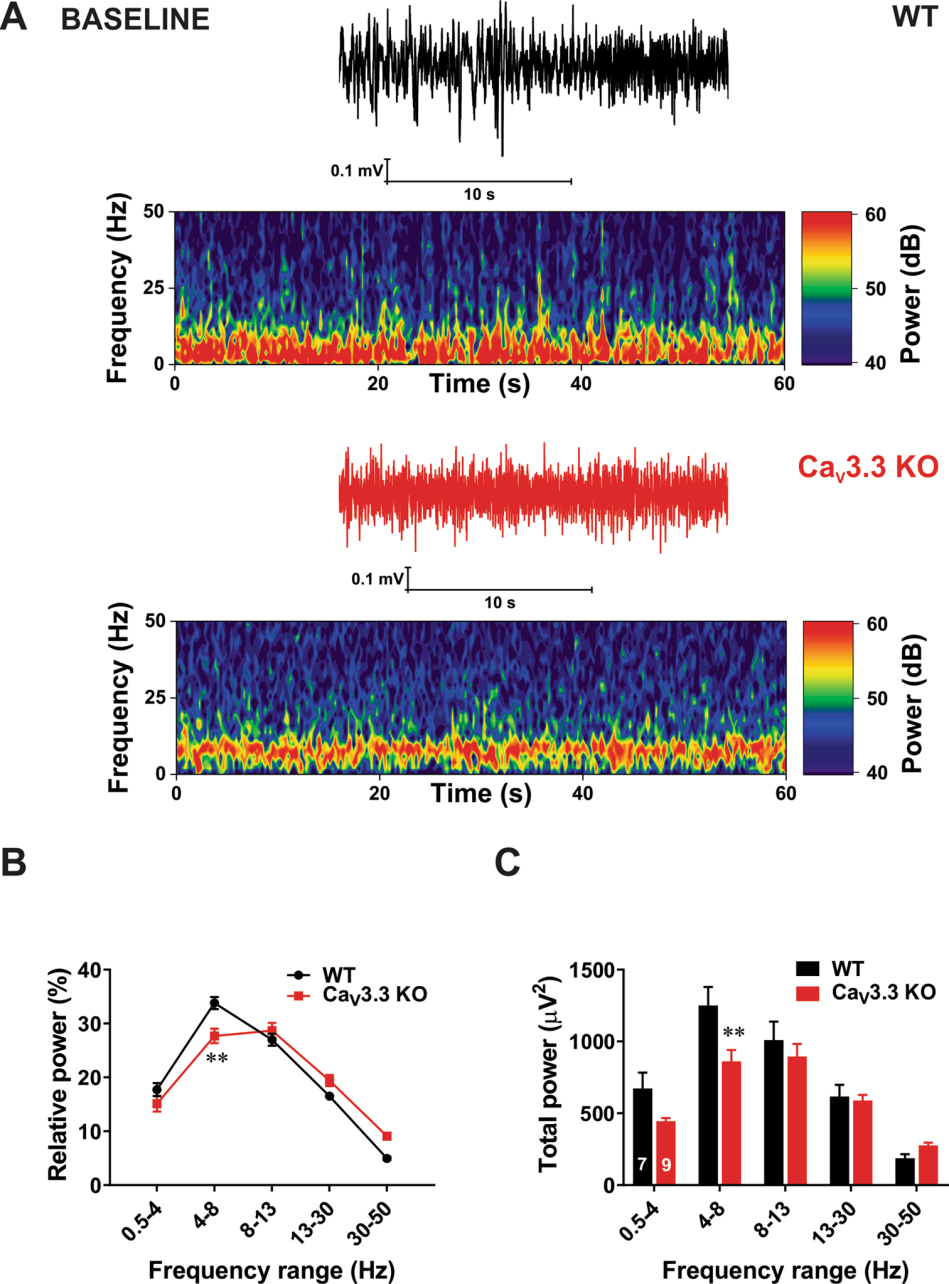
Baseline oscillatory difference between the WT and Ca_V_3.3 KO mice. (**A**) Traces and heat maps from a representative WT mouse (upper panel) and a Ca_V_3.3 KO mouse (lower panel) during quiet awake state. (**B**) Relative power baseline in WT and mutant mice reveled differences in θ range (two-way RM ANOVA: Interaction F_4,56_ = 5.65, *p* < 0.001, Frequency F_4,56_ = 112.80, *p* < 0.001, Strain F_1,14_ = 3.31, *p* = 0.09; Bonferroni post hoc was presented with **p = 0.001). (**C**) Analysis of total power showed increase in slow frequency range (θ range) in the WT mice in comparison with the WT group (two-way RM ANOVA: Interaction F_4,56_ = 6.65, *p* < 0.001, frequency F_4,56_ = 85.61, *p* < 0.001, Strain F_1,14_ = 2.17, *p* = 0.163; Bonferroni post hoc was presented on figure with **p = 0.004). All images were generated using GraphPadPrism8 software (https://www.graphpad.com/scientific-software/prism/).

We first investigated thalamocortical oscillations in vivo in two genotypes during quiet wakefulness before any drug administration. Figure 4A on the top shows representative traces (black colour) and heat plots from a representative WT mouse and bottom trace (red colour) and heat plot are taken from a representative mutant mouse. The relative percent (%) power is calculated for different frequency ranges: δ (0.5–4 Hz), θ (4–8 Hz), α (8–13 Hz), β (13–30 Hz) and low γ (30–50 Hz). Average graph of relative EEG power on Fig. 4B shows significantly diminished oscillations in θ range in KO mice of about 20% when compared to the WT mice, while other frequencies were not affected. Consistent with this finding, Fig. 4C demonstrates that total θ band power was decreased in KO mice for about 30% when compared to the WT mice.

**Figure 5:**
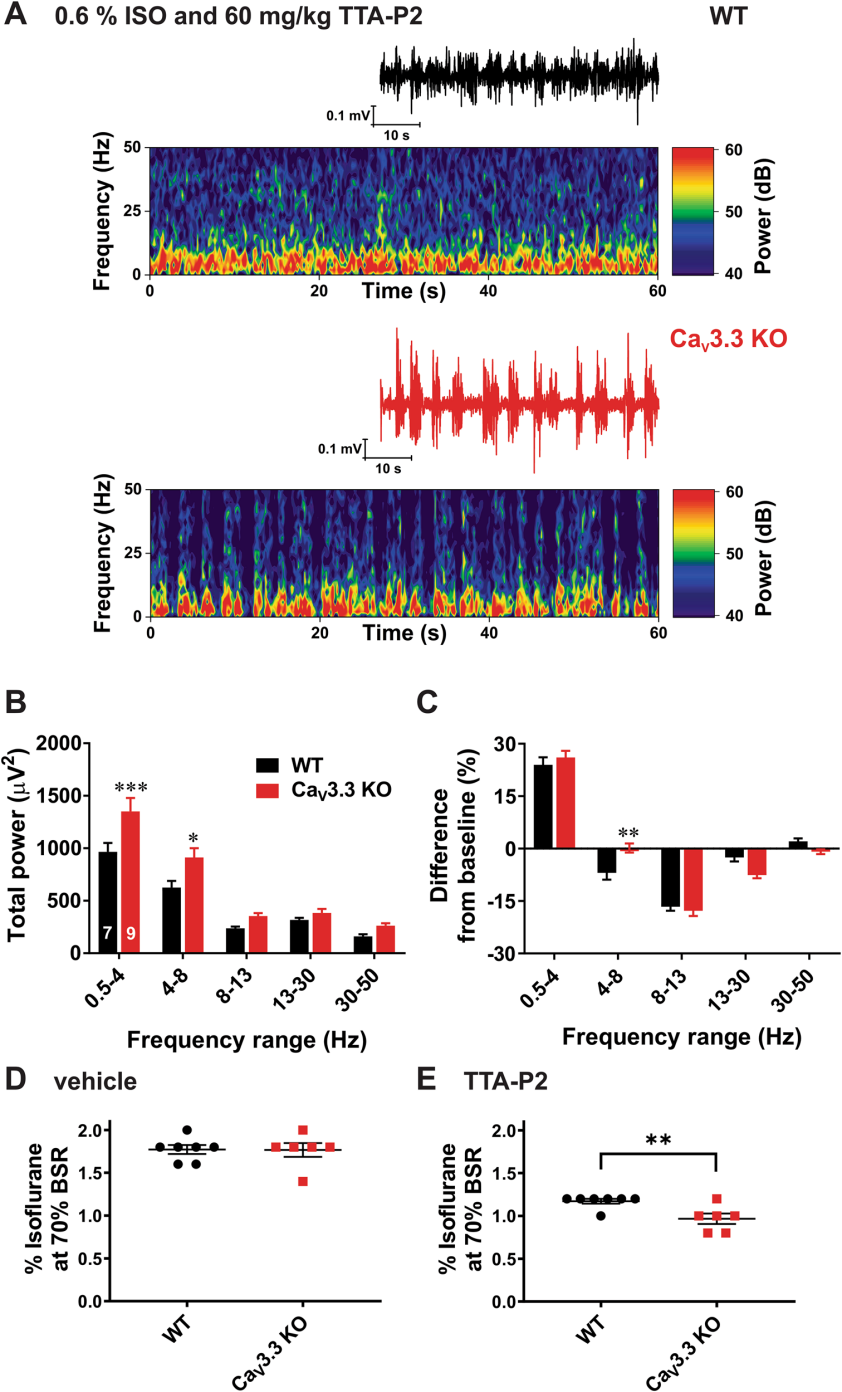
Oscillatory differences between the WT and Ca_V_3.3 KO mice pretreated with TTA-P2 during administration of sub-hypnotic concentrations of isoflurane. (**A**) Representative EEG traces and heat maps from a WT mouse (upper panel) and a Ca_V_3.3 KO mouse (lower panel) during administration of 0.6% isoflurane (ISO) following pretreatment with TTA-P2 at 60 mg/kg i.p. (**B**) Analysis of total power showed a rise in slow frequency range (δ and θ range) in the mutant mice in comparison with the WT group (two-way RM ANOVA: Interaction F_4,56_ = 4.11, *p* = 0.005, Frequency F_4,56_ = 137.40, *p* < 0.001, Strain F_1,14_ = 6.89, *p* = 0.02; Bonferroni post hoc was presented on figure where ***p = 0.0007, and *p = 0.017). (**C**) Relative power during 0.6% isoflurane following pretreatment with TTA-P2 at 60 mg/kg i.p in WT and mutant mice relative to power during wakefulness (two-way RM ANOVA: Interaction F_4,56_ = 4.41, *p* = 0.004, Frequency F_4,56_ = 188.20, *p* < 0.001, Strain F_1,14_ = 0.26, *p* = 0.615; Bonferroni post hoc was presented on figure where **p = 0.004). (**D**) Animals from two cohorts pretreated with 15% cyclodextrin did not show difference in isoflurane requirements to achieve 70% BSR (unpaired two-tailed t-test: t_11_ = 0.051, *p* = 0.960). E) Mutant animals pretreated with 60 mg/kg TTA-P2 achieve 70% BSR with significantly lower isoflurane concentration than the WT group (unpaired two-tailed t-test: t_11_ = 3.177, **p = 0.009). All images were generated using GraphPadPrism8 software (https://www.graphpad.com/scientific-software/prism/).

We next investigated underlying thalamocortical oscillations during combined administration of TTA-P2 and isoflurane. Figure 5A shows representative traces and EEG heat plots during administration of 0.6% isoflurane supplemented with 60 mg/kg i.p. of TTA-P2 from a representative WT (top panels) and a mutant mouse (bottom panels). We found that in KO mice there was a significant increase in the total power frequency ranges δ band and θ band of about 40% when compared to the WT mice (Fig. 5B). Furthermore, when we compared relative EEG power during combined administration of TTA-P2 and isoflurane to the baseline EEG before induction, we found significant difference only in θ band (Fig. 5C).

### TTA-P2 reduced percent of isoflurane required to induce burst suppression EEG pattern more prominently in the Ca_V_3.3 KO mice when compared to the WT mice

Burst-suppression ratio (BSR) has been one of the traditional assays for assessment of depth of anaesthesia, often expressed as 70% BSR^17^. Although baseline BSR values were not different, we noted that both WT and KO mice required significantly less isoflurane to achieve 70% BSR with 60 mg/kg TTA-P2 when compared to the vehicle (Fig. 5D, E, respectively). Namely, WT and Ca_V_3.3 KO mice both demonstrated biological significance at a 33% reduction and a 45% reduction, respectively when comparing the vehicle with TTA-P2 injected groups. Note that KO mice required significantly less isoflurane compared to WT mice when TTA-P2 is administered (Fig. 5E).

Together, this data identifies that the same quality and depth of anaesthesia could be attained with less isoflurane when TTA-P2 was injected, suggesting that T-channel blockers can be used as adjuncts to general anaesthesia. Additionally, this data reveals that the deletion of the Ca_V_3.3 isoform significantly reduces the isoflurane required to reach the same depth of EEG anaesthesia when administered with TTA-P2.

### Ca_V_3.3 KO mice show more prominent isoflurane-sparing hypnotic effect than WT mice in the presence of TTA-P2

**Figure 6:**
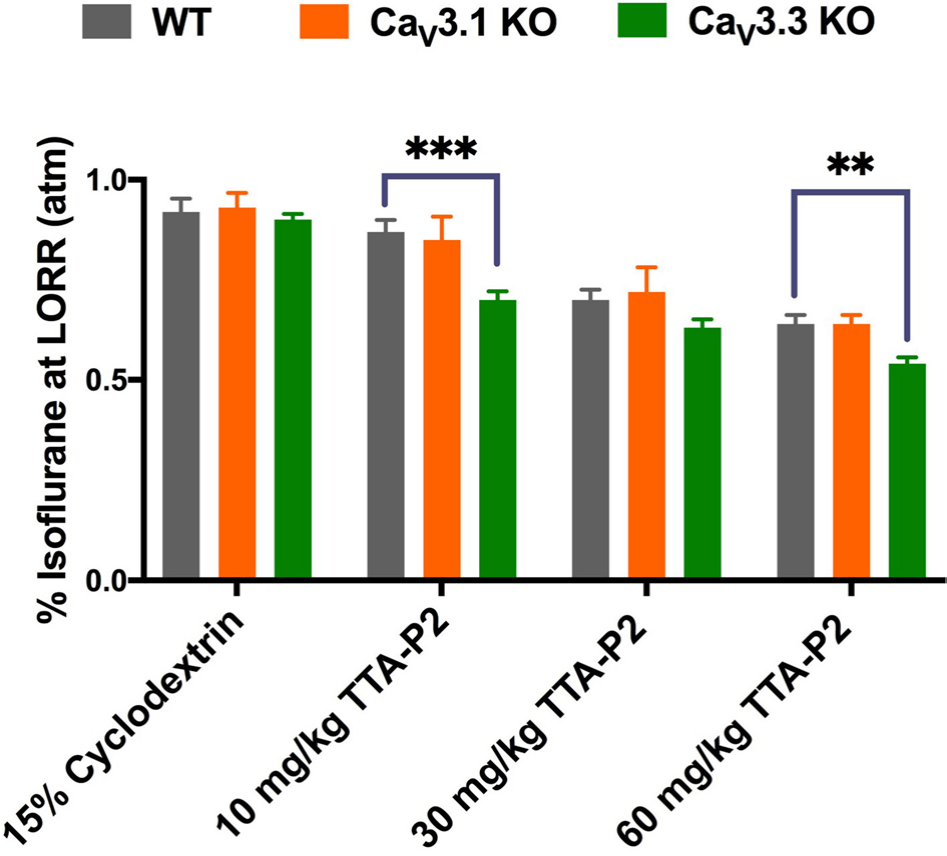
Isoflurane-sparing effect of anaesthetic hypnosis for TTA-P2 is more prominent in the Ca_V_3.3 KO mice than in the WT and Ca_V_3.1 KO mice. The Ca_V_3.3 KO mice pretreated with TTA-P2 required a significantly lower concentration of isoflurane compared to the WT mice at 10 mg/kg and 60 mg/kg of TTA-P2 for each genotype (two-way RM ANOVA: Interaction F_6,81_ = 2.53, *p* = 0.005, Dose F_3,76_ = 127.40, *p* < 0.001, Genotype F_2,27_ = 3.50, *p* = 0.044; Bonferroni post hoc was presented on this figure where ***p < 0.001, and **p = 0.004). In contrast, the Ca_V_3.1 KO mice did not demonstrate a significant difference in response to isoflurane and TTA-P2 when compared to the WT mice. The data for WT and Ca_V_3.3 KO groups were taken from Fig. 3A, B, respectively.

Based on the above EEG data, we predicted that escalating doses of TTA-P2 given i.p. may decrease the percent of isoflurane required to attain LORR in the Ca_V_3.3 KO mice more prominently than in the WT cohort. Hence, we compared the same data presented on Fig. 3A, B. Indeed, we found that the Ca_V_3.3 KO mice were significantly more sensitive to the isoflurane-induced hypnosis as evidenced by decreased amount of isoflurane to reach LORR of about 20% and 15% when compared to the WT mice in the presence of TTA-P2 (10 and 60 mg/kg, respectively; Fig. 6). In contrast, the same anaesthetic protocol did not demonstrate significant difference between the WT and Ca_V_3.1 KO mice. Although we recently reported that Ca_V_3.1 isoform is important for isoflurane-induced thalamo-cortical oscillations^3^, these data strongly suggest that prominent isoflurane-sparing effect of TTA-P2 is relatively specific for the Ca_V_3.3 KO mice.

Finally, to determine if similar isoflurane-sparing effects in hypnosis may be seen with other structurally-unrelated T-channel bockers such as neurosteroids, we pretreated mice with i.p. injections of 3β-OH [(3β,5β,17β)-3-hydroxyandrostane-17-carbonitrile] at 20 mg/kg. At this dose, 3β-OH did not induce LORR when given alone^15^. Although the effect was less prominent when compared to TTA-P2, we found that Ca_V_3.3 KO mice were significantly more sensitive to isoflurane-induced hypnosis than the WT mice when pretreated with 3β-OH (Supplemental Fig. 3).

## Discussion

Our study demonstrates that global deletion of Ca_V_3.3 channels facilitates induction with isoflurane as evidenced by faster TTLORR when compared to the WT mice, while the requirements of isoflurane for the LORR and LOWR were not affected. Interestingly, a low dose of TTA-P2 in WT mice also decreased TTLORR but did not affect LORR. However, when TTA-P2 was administered prior to isoflurane induction, it decreased TTLORR more prominently in the mutant mice when compared to the WT mice. Additionally, we found that TTA-P2 sparing effect for isoflurane-induced hypnosis measured with LORR were stronger in the Ca_V_3.3 KO mice than in the WT and the Ca_V_3.1 KO group. This points to a relatively specific role of Ca_V_3.3 isoform in anaesthetic mechanisms.

An old theory proposed that nonspecific alteration of the lipid membrane in nerve cells accounts for the anaesthetic state^18,19^. However, recent research advances have refuted this idea^20^ and suggested that simultaneous modulation of several membrane proteins contribute to the mechanisms of anaesthesia^21^. Consistent with this view, studies using mouse genetics have demonstrated variable, typically small alterations in effects of GAs^1,22,23^. Although both high-voltage-activated (HVA) and low-voltage-activated (LVA or T-channels) families of voltage-gated calcium channels (VGCCs) have been suggested as possible relevant targets for GAs, precise mechanisms are not well understood. Importantly, T-channels are abundantly expressed in the thalamus where they regulate neuronal excitability. It is well known that most thalamic neurons may fire action potentials in both tonic and post-inhibitory rebound burst firing modes. Tonic firing dominates during awake states, while burst firing is predominant during decreased arousal and sleep. Furthermore, it has been well established that Ca_V_3.3T-channels in nRT are crucial contributors to burst firing mode because during hyperpolarization more T-channels recover from inactivation and may be readily activated during return to resting membrane potentials. Indeed, previous studies with Ca_V_3.3 KO mice reported largely decreased rebound bursting in nRT neurons^14,24,25^. At more depolarized membrane potentials, when most of the T-channels are inactivated, the tonic firing mode is the predominant form of spike discharge.

Biophysical properties of Ca_V_3.3 channels are distinctly different from other T-channel isoforms in that they display two–threefold slower macroscopic inactivation kinetics, which in turn allows for a long lasting burst of action potentials^10^. Specifically, Ca_V_3.3 channels are abundantly expressed on the dendrites of nRT neurons as demonstrated in our cell-attach patch-clamp recordings^12^, by using immunocytochemistry^8^, and optical measurements of dendritic calcium transients^24^. This is important since dendritic arbors are critical for information processing and synaptic integration in thalamic neurons. We have previously demonstrated that GAs preferentially inhibit dendritic vs. somatic T-currents in nRT neurons and proposed that this may contribute to anaesthesia-induced hypnosis^12^. Our current study supports this concept. However, it must be noted that different compensatory mechanisms in KO mice may exist to balance the excitability in thalamocortical circuitry in order to maintain a function as vital as sleep homeostasis. For example, one study found that Ca_V_3.3 KO mice displayed a compensatory increase in tonic firing in nRT neurons, and consequently increased inhibitory synaptic output into TC relay neurons^25^. Furthermore, we have reported increased tonic firing of central medial thalamic nucleus in Ca_V_3.1 KO mice and upregulation of Ca_V_2.3 R-type of VGCCs^3^. Conversely, others have reported upregulation of thalamic Ca_V_3.1 T-currents in mice with global deletion of Ca_V_2.1 genes that encode for P/Q subtype of VGCCs^26,27^. Further work is necessary to investigate other possible mechanisms of homeostatic plasticity in thalamocortical networks in Ca_V_3.3 KO mice. Although it is reassuring that our data with KO mice are mimicked by systemic administration of TTA-P2, targeted tissue-specific gene silencing using viral vectors^25^ or local intrathalamic microdilysis of TTA-P2^28^ could be used in future studies. However, such an approach may be very challenging in the nRT due to its characteristic shell-like structure.

Although finding a unique mechanism of hypnosis is difficult, it appears that the rise in δ frequency cortical oscillations may be consistent across hypnotic states produced by various classes of GAs^28,29^. Indeed, we demonstrate here that a combination of TTA-P2 and subhypnotic concentrations of isoflurane induced more prominent slow wave oscillations in δ range in the mutant mice. Previous studies with Ca_V_3.3 KO mice have established that burst firing on nRT neurons is required for generation of spindle oscillations (7–14 Hz) during slow-wave sleep^14,30^. Although T-channels in nRT neurons alone are sufficient for the rhythmicity of spindles, they are not sufficient for synchrony across the thalamus and cortex^30^. For example, interaction between T-current and I_h_ current in thalamocortical networks is likely to underlie δ oscillations^30,31^. Furthermore, it is generally accepted that slow δ oscillations are a shared feature of sleep and early stages of hypnosis induced by different classes of GAs^28,32^. In contrast, burst suppression is a characteristic EEG pattern of deeper level of anaesthetic hypnosis. It consists of suppression phase—silenced cortical activity and burst phase—episodic high amplitude oscillations. It has been shown that functional or anatomical impairment of cortical afferents leads to the burst suppression pattern^33,34^. During burst suppression, cortical neurons become depolarized during burst episodes and hyperpolarized during silent periods, while some thalamic neurons may exhibit δ rhythm during silent-hyperpolarized states^35^. We found that TTA-P2 facilitated burst suppression in both mutant and WT mice, but the burst suppression pattern was seen with a lower concentration of isoflurane in mutant mice. The facilitatory effects of Ca_V_3.3 deletion on burst suppression may be related to the fact that excitatory synaptic input from the cortex converges to dendrites of nRT where Ca_V_3.3 channels shape dendritic integration. Indeed, previous studies using Ca_V_3.3 KO mice^36^ and optogenetics^37^ have demonstrated that Ca_V_3.3 channels are essential for glutamate-mediated synaptic plasticity in cortico-nRT synapse. Furthermore, because systemic administration of TTA-P2 further facilitated BSR in Ca_V_3.3 KO mice, this confirms that other isoforms of T-channels (e.g. Ca_V_3.1)^3^ contribute as well.

In contrast to δ rhythms and burst suppression, the role of θ oscillations in the mechanisms of GA-induced hypnosis is not well studied. θ oscillations can be generated in both cortical and subcortical structures and are typically present during active awake states and paradoxical sleep, and only briefly before the onset of spindle waves^38^. Furthermore, modeling studies have proposed that thalamic θ oscillations can arise from divergent connections between nRT and non-specific midline thalamic nuclei that provide synchronization of the oscillating circuit^39^. It was reported that administration of subanaesthetic ketamine during isoflurane hypnosis decreases δ power, but increases power of θ, β and γ oscillations^40^. In contrast, we observed simultaneous increase in θ and δ bands during combined administration of TTA-P2 and subanaesthetic isoflurane in mutant mice (Fig. 5). This points to a relatively specific EEG signature of isoflurane-induced hypnosis in Ca_V_3.3 KO mice.

We also found that Ca_V_3.3 KO mice have decreased baseline θ oscillations during wakefulness. Additionally, molecular studies demonstrated rich expression of Ca_V_3.3 channels in the cerebral cortex^5,14^. Our results indicate that Ca_V_3.3 channels contribute to wake θ oscillations in the mouse barrel cortex (present study) but not in frontal and parietal cortex^14^.

In conclusion, our findings strongly suggest the specific value of the Ca_V_3.3 channels in anaesthetic-induced hypnosis. We propose that T-channel blockers may be further explored as a valuable adjunct to reduce the usage of potent volatile anaesthetics, thereby improving their safety. We posit that the potential use of T-channel blockers in clinical anaesthesia warrants further investigation.

## Supplementary information

Supplementary information.
