# Supplementary material for: Global genetic deletion of CaV3.3 channels facilitates anaesthetic induction and enhances isoflurane-sparing effects of T-type calcium channel blockers
Source: Sci Rep. 2020 Dec 9;10:21510. doi: 10.1038/s41598-020-78488-8 (PMC7725806; doi:10.1038/s41598-020-78488-8)
Supplement: Supplementary file 1 — Supplementary information. [file 41598_2020_78488_MOESM1_ESM.pdf]

Global genetic deletion of $Ca_V3.3$ channels facilitates anaesthetic induction and enhances isoflurane-sparing effects of T-type calcium channel blockers

Simon Feseha[1], Tamara Timic Stamenic[1], Damon Wallace[1], Caesare Tamag[1], Lingling Yang[4] Jen Q. Pan[4], Slobodan M. Todorovic[1,2,3]

[1]Department of Anesthesiology, University of Colorado, Anschutz Medical Campus, Aurora 80045; [2]Neuroscience and [3]Pharmacology Graduate Programs, University of Colorado, Anschutz Medical Campus, Aurora 80045; [4]Stanley Center for Psychiatric Research, Broad Institute of Harvard and MIT

✉ Correspondence Author: Slobodan M. Todorovic
Phone 303-724-9122; Fax 303-724-9752
E-mail: slobodan.todorovic@cuanschutz.edu
University of Colorado Anschutz Medical Campus
Department of Anesthesiology, Mail Stop 8130
12801 E. 17th Avenue, Rm L18-4100
Aurora, CO 80045

Running title: the role of $Ca_V3.3$ channels in anaesthesia
Key words: low-voltage-activated, calcium, thalamus, burst suppression, GABA

Acknowledgements
This study was funded in part by grants from the National Institutes of Health (GRANT# R01GM102525 to S.M.T.) and funds from the Department of Anesthesiology and School of Medicine at Anschutz Medical Campus. We thank the University of Colorado Anschutz Medical Campus Rodent *In Vivo* Neurophysiology Core, which is partly supported by the NIH P30 NS048154 grant, for providing facilities to acquire and review video-EEG data.

Conflict of interest
The authors received no compensation, nor do they have any conflicting financial interests with regard to the work described in this manuscript.

SUPPLEMENTAL FIGURES

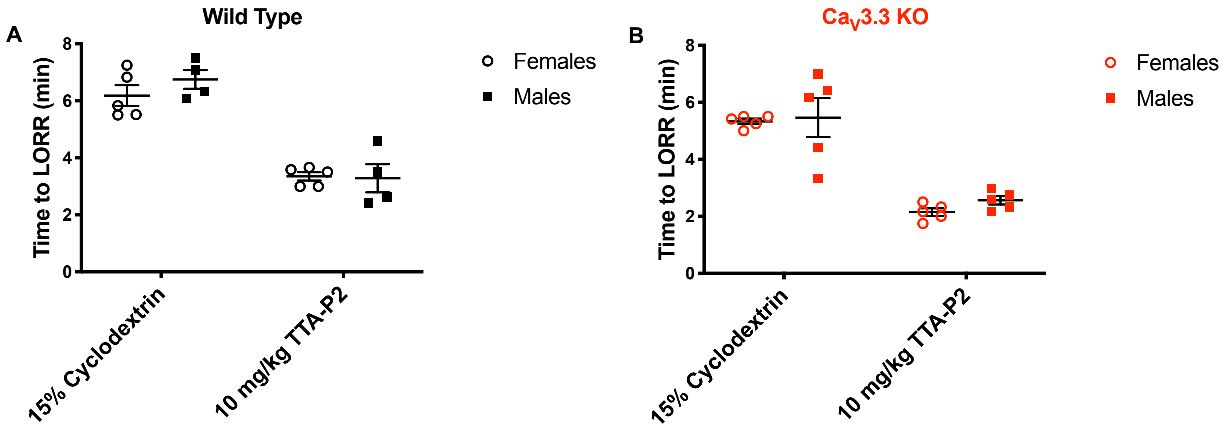

Supplemental Figure 1: A) Time of induction at 1.2% isoflurane comparing male and female mice in the WT mice. ANOVA demonstrated no significance between sexes (two-way repeated measure (RM) ANOVA: $F_{1,7} = 0.82$ p=0.394). Males and females were grouped together for Figure 1A. B) Time of induction at 1.2% isoflurane comparing male and female mice in the $Ca_V3.3$ KO group. ANOVA demonstrated no significance between sexes (two-way repeated measure (RM) ANOVA: $F_{1,8} = 0.79$ p=0.399). Males and females were grouped together for Figure 1A.

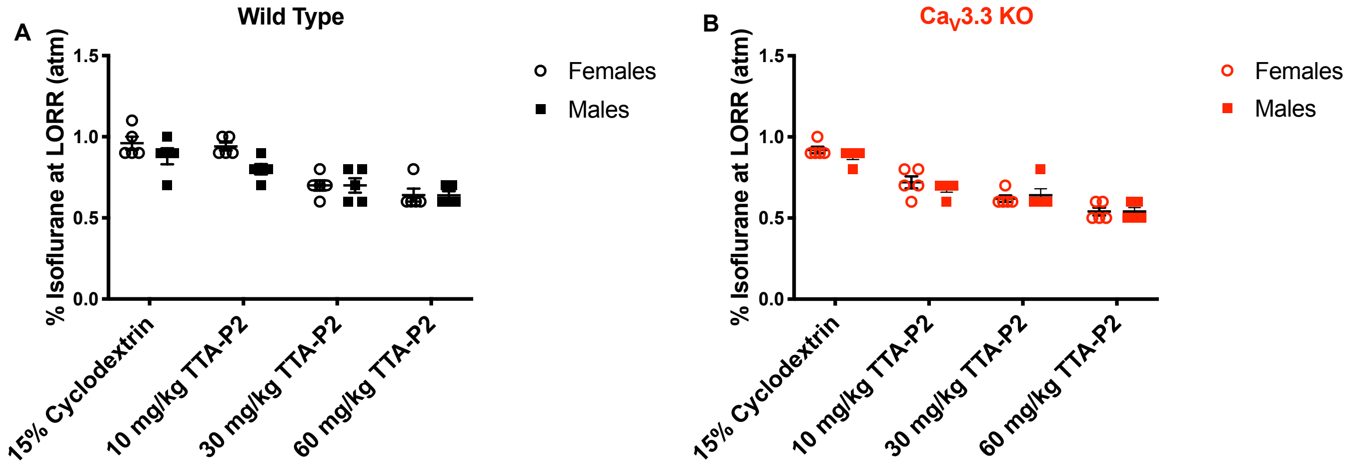

Supplemental Figure 2: A) Percent isoflurane at LORR comparing male and female mice in the WT group. ANOVA demonstrated no significance between sexes (two-way repeated measure (RM) ANOVA: $F_{1,8} = 2.847$ p=0.130). Males and females were grouped together for Figure 3A. B) Percent isoflurane at LORR comparing male and female mice in the Ca$_V$3.3 KO group. ANOVA demonstrated no significant differences between sexes (two-way repeated measure (RM) ANOVA: $F_{1,8} = 0.265$ p=0.621). Males and females were grouped together for Figure 3B.

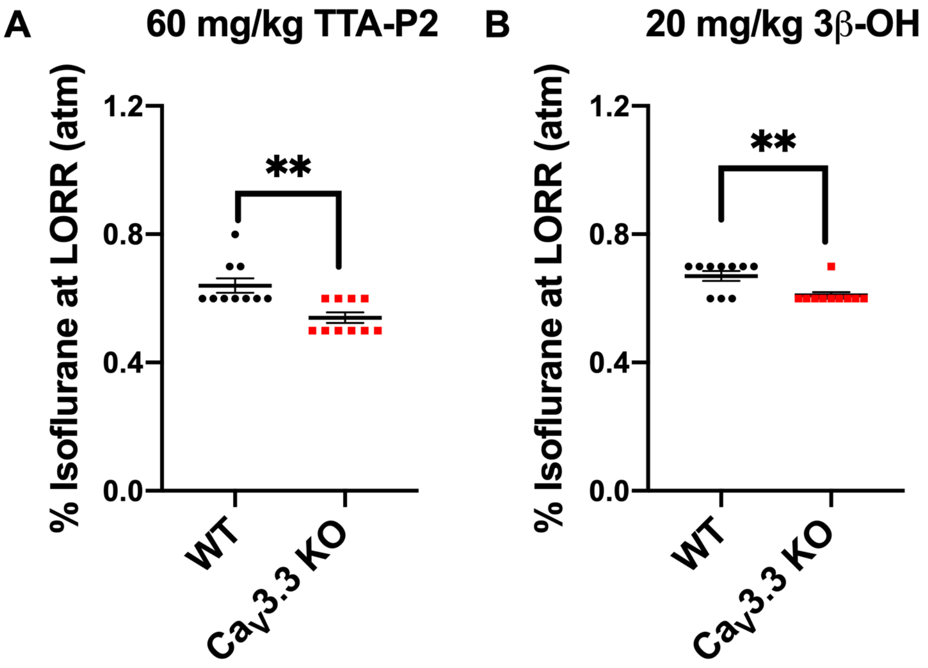

<u>Supplemental Figure 3</u>: Isoflurane-sparing effect of TTA-P2 and 3β-OH on anaesthetic hypnosis is more prominent in the $Ca_V3.3$ KO mice than in the WT mice. A) The $Ca_V3.3$ KO mice pretreated with 60 mg/kg i.p. of TTA-P2 achieved LORR with significantly lower concentration of isoflurane than the WT cohort (unpaired two-tailed t-test: t_{18} = 3.638, ** indicates p = 0.002). The data are taken from Figure 3A and used here as a reference. B) Mutant mice pretreated with 20 mg/kg i.p. of 3β-OH reached LORR at significantly lower concentration of isoflurane that WT mice (unpaired two-tailed t-test: t_{18} = 3.286, ** shows p = 0.004).
